# Vitamin B_12_, Folate, Homocysteine, and Bone Health in Adults and Elderly People: A Systematic Review with Meta-Analyses

**DOI:** 10.1155/2013/486186

**Published:** 2013-02-20

**Authors:** J. P. van Wijngaarden, E. L. Doets, A. Szczecińska, O. W. Souverein, M. E. Duffy, C. Dullemeijer, A. E. J. M. Cavelaars, B. Pietruszka, P. van't Veer, A. Brzozowska, R. A. M. Dhonukshe-Rutten, C. P. G. M. de Groot

**Affiliations:** ^1^Division of Human Nutrition, Wageningen University, P.O. Box 8129, 6700 EV Wageningen, The Netherlands; ^2^Department of Human Nutrition, Warsaw University of Life Sciences (SGGW), Nowoursynowska 159c, 02 776 Warsaw, Poland; ^3^Northern Ireland Centre for Food and Health (NICHE), University of Ulster, Coleraine BT52 1SA, UK

## Abstract

Elevated homocysteine levels and low vitamin B_12_ and folate levels have been associated with deteriorated bone health. This systematic literature review with dose-response meta-analyses summarizes the available scientific evidence on associations of vitamin B_12_, folate, and homocysteine status with fractures and bone mineral density (BMD). Twenty-seven eligible cross-sectional (*n* = 14) and prospective (*n* = 13) observational studies and one RCT were identified. Meta-analysis on four prospective studies including 7475 people showed a modest decrease in fracture risk of 4% per 50 pmol/L increase in vitamin B_12_ levels, which was borderline significant (RR = 0.96, 95% CI = 0.92 to 1.00). Meta-analysis of eight studies including 11511 people showed an increased fracture risk of 4% per **μ**mol/L increase in homocysteine concentration (RR = 1.04, 95% CI = 1.02 to 1.07). We could not draw a conclusion regarding folate levels and fracture risk, as too few studies investigated this association. Meta-analyses regarding vitamin B_12_, folate and homocysteine levels, and BMD were possible in female populations only and showed no associations. Results from studies regarding BMD that could not be included in the meta-analyses were not univocal.

## 1. Introduction 

Osteoporosis is a chronic, multifactorial disorder which is characterized by low bone mass and microarchitectural deterioration of bone tissue [1]. Its major consequence is fractures. Especially hip fractures are frequently associated with institutionalization and increased mortality, and thus with an increased social and economic burden. This burden is expected to increase substantially in Europe in the coming decades due to a rise in life expectancy [2].

Elevated homocysteine concentrations and low vitamin B_12_ and folate status have been associated in several studies with lower bone mineral density (BMD) and higher fracture risk in elderly [3–11].

An elevated plasma homocysteine level (>15 *μ*mol/L) is prevalent in 30–50% of people older than 60 years [12–14]. The cause is multifactorial; a combination of environmental and genetic factors, nutrition, lifestyle, and hormonal factors [15]. Vitamin B_12_ and folate are major determinants of homocysteine metabolism [16, 17] and supplementation with vitamin B_12_ and folic acid has been shown to be effective in normalizing homocysteine levels [18, 19]. Reversing elevated homocysteine levels through folic acid and vitamin B_12_ supplementation could theoretically prevent the problem of impaired bone health and osteoporosis. However, at present, no consensus is reached on the magnitude of the association between vitamin B_12_, folate, homocysteine, and bone health nor on the possible effect of vitamin B_12_ and folate supplementation on bone health.

Up until now one systematic review including a meta-analysis summarized the evidence on homocysteine and fracture risk, showing that higher homocysteine levels significantly increase the risk of fracture [20]. No meta-analyses are known on the topic of folate and vitamin B_12_ in relation to bone health. The purpose of this review is to provide a systematic overview, where possible including pooled estimates of the dose-response association, of the scientific evidence available from randomized controlled trials (RCTs), prospective cohort, and cross-sectional studies addressing vitamin B_12_, folate, and homocysteine levels in association with bone health, that is, fracture risk and BMD, in adults and elderly people. 

## 2. Methods

This systematic review with dose-response meta-analyses was conducted within the scope of the EURRECA (European Micronutrient Recommendations Aligned) Network of Excellence (http://www.eurreca.org/) [21]. We followed a standardized methodology which is described in short below.

### 2.1. Search Strategy and Selection of Articles

We conducted systematic literature searches for (1) vitamin B_12_, (2) folate, and (3) homocysteine. The electronic databases MEDLINE, EMBASE, and Cochrane Library Central were searched, using search terms in “MeSH” terms and “title” and “abstract” on study designs in humans, vitamin B_12_, folate, homocysteine, and intake or status. The full Medline search strategy is available online, (see Appendix 1 in Supplementary Material at http://dx.doi.org/10.1155/2013/486186). 

To be able to use the same search to identify publications on other health related outcomes both in adults and elderly and in younger population groups, no terms were added to limit the search to health outcome or study population. Moreover, by using a broad search we expected a more complete retrieval of relevant publications. In this review only the results on vitamin B_12_, folate, and homocysteine status (i.e., biomarkers measured in serum or plasma) in relation to bone health indicators (fracture risk and BMD) are presented. In addition to the search, reference lists of 10 review articles were checked to identify potentially relevant references that were not identified with the multidatabase search. The search was not limited by language. This review contains studies up to July 2012.

We selected articles in two steps. The first selection step included screening for title and abstract by three independent investigators (J. P. van Wijngaarden, E. L. Doets, SB). In the second selection step, full texts of the selected abstracts were evaluated on basis of predefined inclusion criteria by four investigators (J. P. van Wijngaarden, E. L. Doets, A. Szczecińska, MP). 

For the purpose of alignment and quality control 10% of the references in each selection step was screened and selected in duplicate by two investigators independently. Results were compared and discrepancies were resolved by unanimous consensus among all investigators. 

Studies were eligible for inclusion if they were conducted in apparently healthy human subjects aged ≥18 y. Furthermore, studies had to report fracture incidence, fracture risk, or bone mineral density (BMD) as a health outcome and had to report baseline data on the outcome measure. 

Observational studies were included if they (1) had a prospective cohort, nested case-control, or cross-sectional design, and (2) addressed serum/plasma concentration of markers indicating vitamin B_12_ status (serum/plasma vitamin B_12_, methylmalonic acid (MMA), holotranscobalamin (holoTC)), folate status (serum/plasma folate or erythrocyte folate), or homocysteine status (serum/plasma homocysteine). Intervention studies were included if they (1) had a randomized controlled trial design, (2) studied the effects of vitamin B_12_ or folic acid supplements, fortified foods or micronutrient intake from natural food sources and included a placebo or untreated comparison group, and (3) had a minimum intervention duration of six months.

### 2.2. Data Extraction and Statistical Analysis

We extracted data for each of the identified studies on population characteristics, study design, assessment of vitamin B_12_, folate and homocysteine status, and fracture risk or bone mineral density. 

Opportunities for meta-analysis were evaluated based on comparability of health outcome and status marker. If less than three comparable studies were available, results were qualitatively described. If three or more comparable studies were available, the results of these individual studies were expressed in a standardized format to allow comparison in the form of a continuous dose-response meta-analysis that pools the regression coefficient (*β*) (SE) from multiple adjusted models. We chose to express association measures for serum/plasma vitamin B_12_ per 50 pmol/L. When *β*s were not reported in the original article, we transformed Relative Risk (RR), Hazard Ratio (HR), or Odds Ratio (OR) to *β*s, using a standardized method [22]. The transformations to obtain *β*s and SEs and statistical analyses were performed using R statistics version 2.9.2 (http://www.R-project.org/), with statistical significance defined as *P* < 0.05. HR and OR were considered as RR because the outcome was relatively rare. If articles reported insufficient data (missing data, inconsistencies, or any other uncertainties), we contacted corresponding authors for additional information.

We calculated summary estimates of comparable studies using random effects meta-analysis. Applying the methods of DerSimonian and Laird, the between study variance was estimated which was used to modify the weights for calculating the summary estimate [23]. Residual heterogeneity between studies was evaluated using *Q*-statistic and *I* ^2^-statistic. 

**Figure 1:**
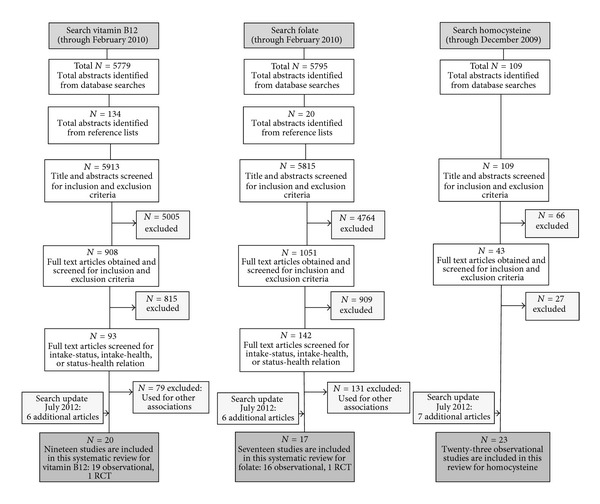
Flow diagram of screening and selection.

In total, from 3 searches we identified 11837 potentially relevant articles, of which 9835 articles were excluded based on title and abstract. Of the remaining 2002 articles, 1961 articles were excluded based on full texts, leaving 41 articles. As the searches were partly overlapping and some articles addressed more than one association this resulted in 20 unique articles, 19 observational and 1 intervention. A search update on July 2nd, 2012 resulted in an additional 8 observational studies, which makes a total of 28 included articles. All addressed the association between vitamin B_12_, folate or homocysteine status, and fracture risk or BMD. The flow diagram of the process of screening and selection is shown in Figure 1.

## 3. Results

### 3.1. Fractures 

#### 3.1.1. Vitamin B_12_

**Figure 2:**
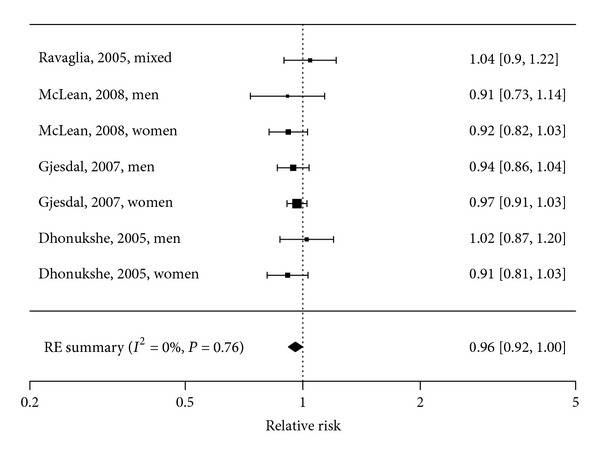
Forest plot of the association between vitamin B_12_ (50 pmol/L) and risk of fracture: Meta-Analysis of 4 observational studies.

Four longitudinal observational studies [3, 24–26], including 7475 elderly people with 3 to 16 years of follow-up and a total of 458 cases addressed the association between serum/plasma vitamin B_12_ and fracture (Table 1). Pooled analysis of the association between 50 pmol/L increase in plasma/serum B_12_ and change in fracture risk showed an inverse association (RR = 0.96, 95%  CI = 0.92 to 1.00) with no heterogeneity between studies (*I* ^2^ = 0%, *P* = 0.76) (Figure 2). This indicates that a vitamin B_12_ increase of 50 pmol/L tends to decrease the risk of fracture with 4%. 

#### 3.1.2. Folate

Three longitudinal observational studies examined the association between plasma folate and fractures [24–26] (Table 2). One study showed that women, but not men, with plasma folate in the lowest quartile had a higher fracture risk (HR 2.40, 95% CI 1.50 to 3.84) compared to the highest (reference) quartile (*P* for trend <0.001) [24]. Ravaglia et al. (2005) [26] showed a significant association between low folate status and fracture risk when folate was analyzed as a dichotomous variable (lowest quartile of folate status versus other 3 quartiles), but when analyzed as a continuous variable, no significant association was observed [26]. One study did not observe an association [25]. 

#### 3.1.3. Homocysteine

**Figure 3:**
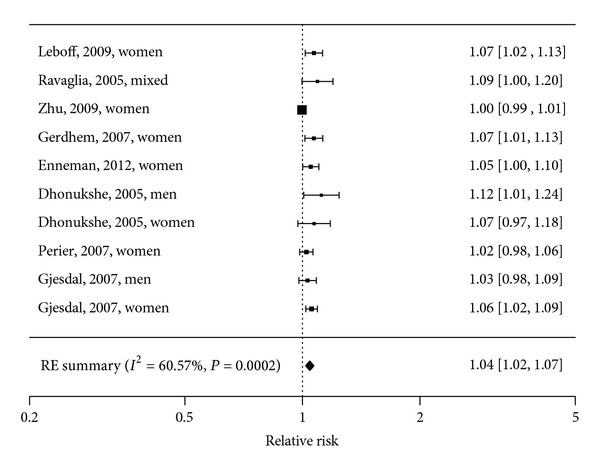
Forest plot of the association between homocysteine and risk of fracture: Meta-Analysis of 8 observational studies.

Eleven longitudinal observational studies examined the association between homocysteine status and fracture incidence [3–5, 25–29] (Table 3). A meta-analysis of eight studies, including 11511 elderly people with 3 to 12.6 years of follow-up and 1353 cases, showed a significantly increased fracture risk with increasing plasma homocysteine (*μ*mol/L) (summary estimate RR 1.04, (95% CI: 1.02 to 1.07). Heterogeneity between studies was large (*I* ^2^ = 60.6%, *P* = 0.0002) (Figure 3). When hip fractures (3 studies; [24, 28, 29]) and total fractures (5 studies; [3, 26, 27, 30, 31]) were analyzed separately, the relation remained significant, 1.06 (95% CI: 1.03 to 1.08, *I* ^2^ = 0%, *P* = 0.72) and 1.04 (95% CI: 1.00 to 1.08, *I* ^2^ = 65.0%, *P* = 0.011). 

Three studies that were not included in the meta-analysis also showed significant associations between homocysteine levels and fracture risk. These studies were not included because the necessary data could not be retrieved from the articles; either homocysteine levels were log-transformed [4, 5] or data was not shown for population homocysteine status [25]. Regardless the type of analysis, women and men in the highest homocysteine quartile had a 1.7 to 3.8 higher RR or HR than those in the lowest or the lowest three quartiles [4, 5, 25]. 

### 3.2. Bone Mineral Density

In the studies included in this review BMD was measured at various sites in the body (e.g., lumbar spine, femoral neck, radius, hip, and total body). As BMD differs per site in the body, we pooled results per biomarker (serum/plasma vitamin B_12_, folate, and homocysteine) and per site for the three sites generally measured (FN, LS, or total hip), thus resulting in 9 meta-analyses. Betas of the individual studies are shown in Tables 1, 2, and 3. The studies included in the meta-analyses took only women into account. Only five studies regarding BMD included a male population [6, 7, 10, 35, 38], and these studies were not comparable quantitatively because differences in the presentation of results or differences in the measured BMD sites.

#### 3.2.1. Vitamin B_12_

Pooled analysis showed no association between serum/plasma vitamin B_12_ levels and BMD in women; FN: *β* = 0.00, 95% CI: −0.13 to 0.14, *I* ^2^ = 0%, *P* = 0.40 [9, 37, 40]; LS: *β* = −2.25, 95% CI: −7.98 to 3.49, *I* ^2^ = 99.5%, *P* < 0.0001 [9, 37, 39, 40]; total hip *β* = −2.23, 95% CI: −10.38 to 5.92, *I* ^2^ = 97.7%, and *P* = 0.0001 [33, 37, 39, 40]. The studies that could not be included in the meta-analyses showed diverse results; in six out of eight studies low serum/plasma vitamin B_12_ was significantly associated with low BMD at at least one site [6, 7, 11, 32, 35, 38]. Two studies did not observe an association between vitamin B_12_ status and BMD [34, 36]. Morris et al. addressed MMA levels as well as a marker for vitamin B_12_ status and observed a lower BMD with higher serum MMA concentrations [7].

#### 3.2.2. Folate

Pooled analysis showed no association between serum/plasma folate and BMD in women; FN: *β* = 0.00, 95% CI: −0.03 to 0.03, *I* ^2^ = 0.00%, *P* = 0.88 [9, 37, 40]; LS: *β* = 0.01, 95% CI: 0.00 to 0.01, *I* ^2^ = 0%, *P* = 0.77 [9, 37, 39, 40]; total hip: *β* = 0.00, 95% CI: 0.00 to 0.01, *I* ^2^ = 78.5%, *P* = 0.0003 [10, 33, 37, 39, 40]. 

From the studies that could not be compared in a meta-analysis, three studies showed significant associations between folate status and BMD or change in BMD over time [8, 10, 34]. Five studies did not observe an association between folate status and BMD [7, 32, 36, 38, 41].

#### 3.2.3. Homocysteine

Pooled analyses showed no association between serum/plasma homocysteine levels and BMD in women; FN: *β* = −0.01, 95% CI: −0.04 to 0.02, *I* ^2^ = 31.5%, *P* = 0.21 [9, 27, 37, 40]; LS: *β* = −0.01, 95% CI: −0.08 to 0.05, *I* ^2^ = 98.4%, *P* < 0.0001 [9, 27, 37, 39, 40]; total hip: *β* = −0.03, 95% CI: −0.08 to 0.02, *I* ^2^ = 99.9%, *P* < 0.0001 [10, 27, 33, 37, 39, 40]. The studies that could not be pooled showed diverse results. In five studies a high homocysteine level was significantly associated with low BMD or change in BMD over time at at least one site [7, 29, 31, 32, 41]. Three studies did not observe a significant association between homocysteine status and BMD or change in BMD [8, 34, 36]. 

### 3.3. Intervention Studies

Up until now, only one RCT (*N* = 47) which met our inclusion criteria studied the efficacy of B-vitamin supplementation on BMD [42]. This study shows some evidence that BMD may be increased with high doses of B-vitamin supplementation in people with hyperhomocysteinemia (tHcy > 15 *μ*mol/L). However, this outcome was only found in a subanalysis of 8 hyperhomocysteinemic subjects [42].

## 4. Discussion

Our meta-analyses showed a significant association of homocysteine levels with fracture risk and a weak though significant inverse association of vitamin B_12_ levels with fracture risk. We could not draw a conclusion regarding folate levels and fracture risk, as too few studies investigated this association. Meta-analyses regarding vitamin B_12_, folate and homocysteine levels and BMD in women found no associations. Results from studies regarding BMD that could not be included in the meta-analyses are not univocal.

 To our knowledge this systematic review with meta-analyses is the most extensive systematic review on the association of vitamin B_12_, folate and homocysteine with bone health until now. Previous non-systematic literature reviews on the association between folate, vitamin B_12_, and homocysteine with bone health reported similar results, that is, conflicting evidence with suggestions towards the association of homocysteine levels with fracture [43–45]. These reviews did not report a systematic literature search strategy and did not provide a quantitative cumulative result. In our review the most recent published articles have been taken into account. The search strategy we used was systematic and extensive, and we used well-defined in- and exclusion criteria. 

One recent systematic review included a meta-analysis on the association between tHcy and fractures [20]. This meta-analysis is different in design than ours, as it is not a dose-response meta-analysis. To overcome the variation in cut-off levels for low vitamin B_12_ and folate status and high homocysteine status, and to allow comparison and subsequent combination of individual studies in the performed meta-analyses, we expressed results of individual studies in a standardized format. We assumed a linear, continuous dose-response association between markers of vitamin B_12_ and folate with fracture rather than a threshold effect. This assumption is generally used in meta-analyses. Furthermore, in some of the key articles addressing the association of homocysteine levels with fractures this association is present [4, 5]. 

A common concern in meta-analyses is heterogeneity between studies. In our meta-analyses we experienced various levels of statistical heterogeneity (no heterogeneity to large heterogeneity). The heterogeneity may be explained by the differences in mean age of the study populations (41–78 years), differences in mean status of vitamin B_12_ (190–549 pmol/L), folate (5.2–24.9 nmol/L) and homocysteine (9.3–16.5 *μ*mol/L), differences in sex distribution of the study populations, duration of follow-up (3–16 years), and level of adjustment for confounders. Although most included studies adjusted for a wide range of confounders for fracture risk or BMD, residual confounding by other unmeasured or inadequately measured factors cannot be ruled out. For example, low vitamin D status is a risk factor for fracture [46]. From the studies included in our meta-analyses for fracture three out of nine adjusted for vitamin D status [24, 25, 27]. Outcomes do not seem to differ between studies that corrected for vitamin D status and studies that did not. Homocysteine levels are increased with renal dysfunction, often measured by serum or urine creatinine levels. Five out of eight studies in the meta-analysis regarding homocysteine and fracture risk corrected for creatinine levels [3, 24, 26, 27, 29], and outcomes did not seem to differ. 

As almost all studies were performed in countries without mandatory folate fortification or were performed before the fortification era in the USA and Australia, we do not consider folate fortification as a source of heterogeneity in our analyses.

The majority of studies included were longitudinal and cross-sectional observational studies. We could only include one intervention study, which had a very small study population (*N* = 47). One intervention study which found a beneficial effect of vitamin B_12_ and folic acid supplementation on fracture risk could not be included in our systematic review, because this study investigated a population of hemiplegic patients following stroke [47]. The generalizability of these findings is confined to a highly selective patient population with a high percentage of vitamin D deficiency and a high fracture risk. As evidence from intervention studies is lacking, currently no causal effect between vitamin B_12_, folate and homocysteine levels and bone health can be established. Consequently, it is yet unknown whether extra vitamin B_12_ and folate intake through supplementation could reverse the observed negative effects of vitamin B_12_ and folate deficiency and elevated homocysteine levels. Further evidence from an intervention study is expected soon, as a large intervention study on the effect of vitamin B_12_ and folic acid supplementation on fracture risk, BMD, and bone turnover markers is currently carried out with results expected in 2013 [48]. 

As the quality of included studies determines the quality of the review and meta-analysis, we assessed the overall risk of bias of each individual study using standardized procedures largely based on guidance from the Cochrane Collaboration [49], resulting in one of the following judgments: low, moderate, or high risk of bias. Twenty out of the 28 included studies were evaluated as having moderate (*n* = 15) or high risk (*n* = 5) of bias. These studies did take one or more of the predefined confounders into account, that is, age, sex, smoking, physical activity, and body weight, or the study was funded or cofunded by a commercial organization. Due to the limited number of studies included in the meta-analyses, we were not able to study the effect of the overall risk of bias, nor of its single components on the pooled effect measures. There seems to be no difference in the outcomes of studies with low risk of bias compared to studies with moderate or high risk of bias, and we therefore assume that the quality of the included studies had no effect on the outcome of this review.

The intake of folate and vitamin B_12_ are a determinant of folate, vitamin B_12_, and homocysteine status. To deal with potential malabsorption of vitamin B_12_ [50] and reduced bioavailability of folate [51], the use of biomarkers for vitamin B_12_ and folate status is preferred over measures of intake when studying associations with bone health in elderly people. 

In studies addressing folate status, serum or plasma folate was measured, which is considered as an appropriate marker for folate status in epidemiological studies [52]. Homocysteine is a nonspecific marker for both folate and vitamin B_12_ status [53], which makes it a relevant biomarker in this review. Regarding the metabolic interactions between vitamin B_12_, folate, and homocysteine combined with the variety in data presented in the studies, we were not able to investigate the possibility that a low vitamin B_12_ or folate status in combination with a high homocysteine level might result in a higher fracture risk in comparison to a low vitamin B_12_ or folate status or homocysteine level alone. In most studies regarding vitamin B_12_ status, status was assessed with serum or plasma vitamin B_12_. Other, more sensitive markers for vitamin B_12_ deficiency, like MMA and HoloTC [54], were addressed only in a few studies. We could therefore not draw conclusions about the association between these biomarkers and outcomes on bone health.

There are several suggested mechanisms for the association between vitamin B_12_, folate, homocysteine, and bone health. Homocysteine may interfere with collagen cross-linking. Cross-links are important for the stability and strength of the collagen network. Interference in cross-link formation would cause an altered bone matrix, resulting in more fragile bones [55]. As collagen cross-links do not alter BMD, this may explain why a more convincing result is found regarding fractures than BMD, as suggested for example by Van Meurs et al. [5]. Vitamin B_12_ deficiency has been associated with impaired functional maturation of osteoblasts [56]. Some *in vitro* studies support the hypothesis of a possible favorable effect of vitamin B_12_ supplementation, although results are equivocal. Vitamin B_12_ has been shown to stimulate osteoblast proliferation and alkaline phosphatase activity [57] but Herrmann et al. were not able to show any significant and consistent effect of vitamin B_12_ or folic acid on osteoblast activity [58]. Recent publications show evidence of osteoclast stimulation in the presence of high homocysteine and low vitamin B_12_ concentrations [59–61]. Vitamin B_12_ and folate are not the only B-vitamins involved in the homocysteine metabolism. Various micronutrients, such as vitamin B_2_ (riboflavin), vitamin B_6_ (pyridoxine), and choline also affect homocysteine levels [16, 17, 62], and may consequently affect bone health. Given that vitamin B_12_ and folate are the main factors influencing homocysteine levels, and therefore the primary focus in a homocysteine lowering intervention [63], our review focused on vitamin B_12_, folate, and homocysteine. 

*Considerations for Future Research and Conclusions*

The mechanisms involved in the association between biomarkers of B-vitamins and bone health are still unclear and therefore more fundamental research is required to establish the potential mechanisms. Subsequently, both observational and intervention studies should preferably not focus on just one biomarker in relation to the homocysteine metabolism, but take a biomarker profile into account, including serum/plasma vitamin B_12_, MMA, HoloTC, folate, and homocysteine levels. Evidence is needed from well-designed, large intervention studies to establish a causal relationship between markers of B-vitamins and bone health.

This systematic review with meta-analyses shows that elevated homocysteine levels are associated with increased fracture risk. Vitamin B_12_ status may be associated with fracture risk and evidence for an association between folate status and fracture risk is scarce. Vitamin B_12_, folate, and homocysteine levels are probably not associated with BMD, but results are not univocal.

## Supplementary Material

The electronic databases MEDLINE, EMBASE, and Cochrane Library Central were searched, using search terms in “MeSH” terms and “title” and “abstract” on study designs in humans, vitamin B_12_, folate, homocysteine, and intake or status. The fullMedline search strategy is available in Appendix 1.

## Figures and Tables

**Table 1:** Studies regarding the association between vitamin B_12_ and bone health.

Author Year	Study characteristics Duration of follow-up (when applicable) Country *Risk of bias *	Population characteristics: N (% men) Age (y) ± SD	Vitamin B_12_ status pmol/L* Mean ± SD	Outcome	Association type		Results*	
Dhonukshe-Rutten et al. 2005 [3]	Cohort (3 y) The Netherlands *High risk *	1253 (48%) 75.5 ± 6.6	♀: 289 ± 99 ♂: 268 ± 89	Fracture (verified by physician or radiograph)	*β* (SE) for association vitB_12_-fracture (per 50 pmol/L)		♀: −0.09 (0.06)^a, 1^ ♂: 0.02 (0.08)^a, 1^	

Gjesdal et al. 2007 [24]	Cohort (12.6 y) Norway *Low risk *	4761 (45%) 65–67 at baseline	♀: 386.4 ± 372.0 ♂: 359.3 ± 276.2	Hip fracture (verified by hospital discharge diagnoses)	*β* (SE) for association vitB_12_-hip fracture (per 50 pmol/L)		♀: −0.03 (0.03)^b, 2^ ♂: −0.06 (0.05)^b, 2^	

McLean et al. 2008 [25]	Cohort (16 y) USA *Low risk *	823 (41%) 75.3 ± 4.9	Deficient (<148): ♀ 9%/♂14.0% Low (148–257.9): ♀ 24.3%/♂32.5% Normal (≥258): ♀ 66.7%/♂53.5%	Hip fracture (verified by review medical records)	*β* (SE) for association vitB_12_-hip fracture (per 50 pmol/L)		♀: −0.09 (0.06)^c, 1^ ♂: −0.09 (0.11)^c, 1^	

Ravaglia et al. 2005 [26]	Cohort (4 y) Italy *Moderate risk *	702 (47%) 73.0 ± 6.0	Geometric mean (95% CI) 249.1 (203–272)	Fracture (verified by review medical records)	*β* (SE) for association vitB_12_-fracture (per 50 pmol/L)		0.04 (0.08)^d, 2^	

Bozkurt et al. 2009 [32]	Cross-sectional Turkey *High risk *	178 (0%) 53.5 ± 8.0	247.7 ± 85.4	BMD: LS, FN [DXA]	Logistic regression for FN, LS and FN + LS combined for vitB_12_ status under the quintile value. *β* (SE) + *P* value		LS: −2.3 (0.9) *P* = 0.017 FN: −0.4 (0.9) *P* = 0.669 LS + FN: 1.8 (0.8) *P* = 0.045^e^	

Bucciarelli et al. 2010 [33]	Cross-sectional Italy *Moderate risk *	446 (0%) 65.1 ± 9.4	(geometric mean ± SD) 399.1 ± 1.6	BMD: FN, LS, TH [DXA, Prodigy, GE, Lunar]	*β* for association vitB_12_-TH BMD *β* (SE) (per 50 pmol/L)		−0.00105 (0.939)^f, 2^	

Cagnacci et al. 2008 [34]	Cohort (5 y) Italy *Moderate risk *	117 (0%) 54.4 ± 0.5	(Mean ± SE) 548.5 ± 40.5	BMD: LS [DXA: Lunar DPX]	Regression for vitB_12_-BMD change *β* (SE) *P* value		−0.003 (0.012) *P* = 0.784^g^	

Dhonukshe-Rutten et al. 2003 [35]	Cross-sectional The Netherlands *Moderate risk *	194 (26%) 78.3 ± 5.5	♀ 288 ± 131 ♂ 238 ± 95	BMD: whole body [DXA, Lunar DPX-L]	Multivariate regression, *β* for association vitB_12_-BMD *β* (95% CI) in women		♀: 12.3·10^−5^ (0.2·10^−5^–2.4·10^−4^)^h^	

Gjesdal et al. 2006 [10]	Cross-sectional Norway *Moderate risk *	5329 (43%) middle aged: 47–50 Older: 71–75	♀ 393.4 ± 235.8 ♂ 374.6 ± 230.7	BMD: TH [DXA, Lunar EXPERT-XL]	OR (95% CI) for low BMD per category vitB_12_ status 1: <230 pmol/L 2: 230.0–279.9 pmol/L 3: 280.0–414.9 pmol/L 4: ≥415.0 pmol/L + *P* for trend		♀: 1: 0.97 (0.68–1.37) 2: 0.87 (0.63–1.21) 3: 1.02 (0.82–1.27) 4: 1.00 (reference) *P* for trend = 0.61	♂: 1.22 (0.82–1.81) 1.14 (0.80–1.62) 0.97 (0.74–1.28) 1.00 (reference) *P* for trend = 0.25^i^

Golbahar et al. 2004 [9]	Cross-sectional Iran *Moderate risk *	271 (0%) 60.8 ± 6.8	(geometric mean ± SD) 339.5 ± 247.6	BMD: FN, LS [DXA, Lunar DPX-L]	*β* (SE) for association vitB_12_-BMD (per 50 pmol/L)		FN: 0.0002 (0.07)^2^ LS: 0.0114 (0.14)^2^	

Haliloglu et al. 2010 [36]	Cross-sectional Turkey *Moderate risk *	120 (0%) 54.4 ± 1.1	Osteoporotic: 216.0 ± 135.1 Osteopenic: 190.8 ± 97.4 Normal BMD: 251.0 ± 205.8	BMD: LS [DXA, Lunar DPX-L]	ANOVA for difference in vitB_12_ status per BMD group compared to normal BMD group		No sign differences in vitB_12_ status between BMD groups	

Krivosikova et al. 2010 [37]	Cross-sectional Slovakia *High risk *	272 (0%) 41.3 ± 19.8	273.2 ± 152.7	BMD: FN, LS, trochanter, TH [DXA, Lunar DPX-L]	Stepwise multivariate linear regression, *β* for association vitB_12_-BMD. *β* (SE) *P* value (per 50 pmol/L)		FN: −2.0 (2.73) ^j, 2^ LS: −1.15 (1.42)^ j, 2^ TH: −0.5 (3.03) ^j, 2^	

Morris et al. 2005 [7]	Cross-sectional USA *Low risk *	1550 (48%) 68	Geometric mean (95% CI) Osteoporosis: 271 (243–302) Osteopenia: 309 (293–325) Normal: 310 (297–323) Serum MMA (nmol/L) Osteoporosis: 305 (276–337) Osteopenia: 251 (234–269) Normal: 241 (212–274)	BMD: Trochanter, intertrochanter, FN, Ward's triangle, TH [DXA, Hologic QDR-1000]	OR (95% CI) for mean BMD in relation to quartile categories of vitB_12_ and MMA status + *P* for trend. Category medians:		Vit B_12_: Q1: 2.0 (1.0–3.9) Q2: 1.3 (0.6–2.7) Q3: 1.7 (0.8–3.3) Q4: 1.0 (reference) *P* for trend = 0.09	MMA: 1.0 (reference) 3.5 (1.4–8.5) 5.2 (2.0–13.1) 7.2 (3.4–15.2) *P* for trend <0.001^k^
					B_12_ (pmol/L): Q1: 182 Q2: 268 Q3: 349 Q4: 495	MMA (nmol/L): 157 206 272 415	Among subjects with vitB_12_ <220 pmol/L mean BMD increased sign with increasing vitB_12_ (*P* = 0.01)	

Naharci et al. 2012 [38]	Cross-sectional Turkey *Moderate risk *	264 (100%) 77.0 ± 6.0	26.7% low (<148, group I) 39.1% borderline (148–221, group II) 34.2% normal (>221, group III)	BMD: FN, TH, trochanter, inter-trochanter [DXA, hologic QDR-4500]	Anova for differences in FN BMD between groups of serum vitB_12_		Sign differences FN BMD group I and II (*P* = 0.013) group I and III (*P* < 0.001) group II and III (*P* = 0.003) FN BMD was positively correlated with serum vitB_12_ (r = 0.362, *P* < 0.001)	

Ouzzif et al. 2012 [39]	Cross-sectional Morocco *Moderate risk *	188 (0%) 57.8 ± 8.5	360.4 ± 149.2	BMD: FN, LS, TH, trochanter [DXA, Lunar prodigy]	Multivariate regression, *β* for association vitB_12_-BMD *β* (SE) (per 50 pmol/L) *P* value		LS: −7.85 (0.25) TH: −11.65 (0.02)	*P* = 0.160^L, 2^ *P* = 0.007^L, 2^

Rumbak et al. 2012 [40]	Cross-sectional Croatia *Low risk *	131 (0%) 54.0 ± 4.9	239.6 ± 97.0	BMD: FN, LS, TH, radius [DXA, Lunar-prodigy]	Stepwise multivariate regression, *β* for association vitB_12_-BMD for pre- and postmenopausal women *β* (SE) *P* value (per 50 pmol/L)		Premenopausal: LS: −3.39 (8.91) *P* = 0.709^m, 2^ FN: 7.45 (10.07) *P* = 0.467^m, 2^ TH: −1.36 (7.53) *P* = 0.862^m, 2^ Postmenopausal: LS: 7.45 (8.98) *P* = 0.411^m, 2^ FN: 12.20 (8.97) *P* = 0.180^m, 2^ TH: 8.81 (8.63) *P* = 0.314^m, 2^	

Stone et al. 2004 [11]	Cohort (5.9 y) USA *Low risk *	83 (0%) 71.1 ± 4.4	352 ± 174	BMD: TH, FN (change) [DXA, Hologic QDR-1000]	*t*-test for difference in BMD change between low and normal vitB_12_ status		Participants with low vitB_12_ (≤207 pmol/L) had a more rapid decline in BMD (−1.91%/year) than part. with normal vitB_12_ (−0.10%/year), *P* < 0.05	

Tucker et al. 2005 [6]	Cross-sectional USA *Low risk *	2576 (44%) 58.8 ± 9.5	Distribution per category of plasma vitB_12_ status 1: ♀ 4.4%/♂4.7% ≤148 2: ♀ 6.9%/♂7.8% >148–185 3: ♀ 25.4%/♂28.2% >185–259 4: ♀ 63.3%/♂ 59.3% >259	BMD: FN, LS, TH, Trochanter, Ward [DXA, Lunar DPX-L]	Differences in BMD per category of plasma vitB_12_ level, relative to category 1		♀: FN: no differences ♀: LS: vitB_12_ in cat 2 (*P* < 0.10), 3, 4 (*P* < 0.05) was assoc. with better BMD ♀: TH: vitB_12_ in cat 3, 4 (*P* < 0.10) was assoc. with better BMD ♂: FN: vitB_12_ in cat 2, 3, 4 was assoc. with better BMD (*P* < 0.05) ♂ LS: no differences ♂ TH: vitB_12_ in cat 2 (*P* < 0.10), 3, 4 (*P* < 0.05) was assoc. with better BMD^n^	

*Serum/plasma vitamin B_12_ concentrations were converted to pmol/L if applicable, using the following equation: 1 pg/mL = 1 ng/L = 0.738 pmol/L. subsequent outcomes were also converted. Where possible, subgroups were combined. BMD sites: LS: Lumbar Spine, FN: Femoral Neck, TH: Total Hip.

^1^ *β*(SE) as calculated from data provided by author; ^2^ *β* (SE) as calculated from presented data.

^a^adjusted for age, BMI, smoking, recurrent falling; ^b^adjusted for age, BMI, smoking, coffee intake, physical activity, vit D use, educational level, estrogen use in women; ^c^adjusted for sex, age, height, weight, estrogen use in women; ^d^adjusted for age, sex, education, osteoporosis drugs, creatinine, tHcy; ^e^adjusted for duration of menopause, smoking, BMI, folic acid levels, tHcy levels; ^f^adjusted for age, BMI, logtHcy, logFolate, creatinine clearance, smoking, alcohol intake; ^g^Adjusted for age, weight, weight change; ^h^adjusted for weight, height, energy intake; ^i^adjusted for smoking, BMI, creatinin, coffee intake, physical activity, use of estrogen therapy; ^j^adjusted for age, folate, tHcy, PTH, CTx, Ca, Cr; ^k^Adjusted for age, sex, ethnicity, BMI, smoking, physical activity, creatinin, alcohol, coffee, energy, calcium, vitamin D zinc intake; ^L^adjusted for age, BMI, tHcy and folate; ^m^adjusted for Age, BMI, smoking, alcohol, physical activity, tHcy, Folate; ^n^adjusted for energy, calcium, vitamin D intake, BMI, height, smoking, age, physical activity, calcium supplement, vitamin D supplement, alcohol, osteoporosis medication, season of measurement.

**Table 2:** Studies regarding the association between folate and bone health.

Author Year	Study characteristics Duration of follow-up (when applicable) Country *Risk of bias *	Population characteristics: *N* (%men) Age (y) ± SD	Folate status (nmol/L)* Mean ± SD	Outcome	Association type	Results*	
Gjesdal et al. 2007 [24]	Cohort (12.6 y) Norway *Low risk *	4761 (45%) 65–67 at baseline	♀ 6.0 ± 3.5 ♂ 5.2 ± 2.7	Hip fracture (verified by hospital discharge diagnoses)	HR for hip fracture according to folate status 1: <2.9 2: 2.9–3.8 3: 3.9–6.5 4: ≥6.6	♀: 1: 2.40 (1.50–3.84) 2: 1.15 (0.68–1.94) 3: 1.02 (0.68–1.54) 4: 1.00 (reference)^a ^	♂: 1.00 (0.48–2.12) 0.80 (0.39–1.62) 0.81 (0.45–1.46) 1.00 (reference)^a^

McLean et al. 2008 [25]	Cohort (16 y) USA *Low risk *	960 (41%) 75.3 ± 4.9	Not shown	Hip fracture (verified by review medical records)	HR for hip fracture according to folate status Normal: ≥11 Low: 7–10.9 Deficient: <7	Normal: 1.00 (reference) Low: 0.76 (0.43, 1.32) Deficient: 1.38 (0.91, 2.09)^b^	

Ravaglia et al. 2005 [26]	Cohort (4 y) Italy *Moderate risk *	702 (47%) 73.0 ± 6.0	11.7 (9.0–12.2) mean (95% CI)	Fracture (verified by review medical records)	OR (95% CI) for risk of fracture at follow-up for each increment of 1 sd in the log-transformed serum folate value	0.83 (0.59–1.19)^c^	

Baines et al. 2007 [41]	Cross-sectional Great Britain *High risk *	328 (0%) 67.5 (40–85) mean (range)	Osteoporosis: 8.1 ± 8.7^#^ Osteopenia: 10.2 ± 4.6 Normal: 9.4 ± 6.3	BMD: os calcis/ heel bone [PIXI, GE Lunar]	ANOVA for difference between the normal, osteopenia and osteoporosis group	FA status was significantly different between osteroporotic and osteopenic group (*P* = 0.049)	

Bozkurt et al. 2009 [32]	Cross-sectional Turkey *High risk *	178 (0%) 53.5 ± 8.0	24.9 ± 7.9	BMD: FN, LS [DXA]	Logistic regression for FN, LS and FN + LS combined. *β* (SE) + *P* value for assoc. BMD-folate status under the median value	LS: −0.2 (0.2) *P* = 0.417 FN: −0.04 (0.2) *P* = 0.835 LS + FN: −0.03 (0.2) *P* = 0.896^d^	

Bucciarelli et al. 2010 [33]	Cross-sectional Italy *Moderate risk *	446 (0%) 65.1 ± 9.4	(geometric mean ± SD) 3.8 ± 1.6	BMD: FN, LS, TH [DXA, Prodigy, GE, Lunar]	*β* for association folate-TH BMD *β* (SE)	0.004 (0.018)^e, 2^	

Cagnacci et al. 2008 [34]	Cohort (5 y) Italy *Moderate risk *	117 (0%) 54.4 ± 0.5	(Mean ± SE) 20.6 ± 1.4	BMD: LS [DXA: Lunar DPX]	Regression analysis for folate-BMD change *β* (SE) + *P* value	1.602 (0.803) *P* = 0.048^f^	

Cagnacci et al. 2003 [8]	Cross-sectional Italy *Moderate risk *	161 (0%) 53.3 ± 1.04	(Mean ± SE) 21.5 ± 4.3	BMD: LS [DXA: Lunar DPX]	Regression analysis, *r* (*P* value) for association folate-BMD	*r* = 0.254	(*P* < 0.002)

Gjesdal et al. 2006 [10]	Cross-sectional Norway *Moderate risk *	5329 (43%) middle aged: 47–50 Older: 71–75	♀ 8.9 ± 7.1 ♂ 7.3 ± 4.6	BMD: TH [DXA, Lunar EXPERT-XL]	OR (95% CI) for low BMD per category folate status: 1: FA < 3.8 nmol/L 2: FA 3.8–4.9 nmol/L 3: FA 5.0–8.4 nmol/L 4: FA ≥ 8.5 nmol/L + *P* for trend Multivariate regression for folate-BMD *β* (SE) (per 50 nmol/L)	♀: 1: 1.55 (1.07–2.23) 2: 1.18 (0.86–1.63) 3: 1.24 (0.99–1.56) 4: 1.00 (reference) *P* for trend = 0.02	♂: 0.81 (0.53–1.24) 0.96 (0.67–1.38) 1.15 (0.87–1.53) 1.00 (reference) *P* for trend = 0.26^g^
						Elderly women: *β* = 0.05 (0.02)^g, 2^	

Golbahar et al. 2004 [9]	Cross-sectional Iran *Moderate risk *	271 (0%) 60.8 ± 6.8	(geometric mean ± SD) 11.6 ± 6.5	BMD: FN, LS [DXA, Lunar DPX-L]	*β* for association folate-BMD *β* (SE)	FN: 0.008 (0.019)^h, 2^ LS: 0.010 (0.018)^i, 2^	

Haliloglu et al. 2010 [36]	Cross-sectional Turkey *Moderate risk *	120 (0%) 54.4 ± 1.1	Osteoporotic: 12.2 ± 6.3 Osteopenic: 15.4 ± 7.4 Normal: 15.8 ± 8.3	BMD: LS [DXA, Lunar DPX-L]	ANOVA for difference in folate status per BMD group (osteoporotic, osteopenic, compared to normal BMD group)	No significant differences in folate status between BMD groups	

Krivosikova et al. 2010 [37]	Cross-sectional Slovakia *High risk *	272 (0%) 41.3 ± 19.8	23.8 ± 9.6	BMD: FN, LS, trochanter, TH [DXA, Lunar DPX-L]	Stepwise multivariate linear regression, *β* for association folate-BMD. *β* (SE) *P* value	FN: −0.028 (0.054) *P* = 0.606 ^j, 2^ LS: −0.001 (0.067) *P* = 0.988 ^j, 2^ TH: −0.032 (0.060) *P* = 0.595^ j, 2^	

Morris et al. 2005 [7]	Cross-sectional USA *Low risk *	1550 (47%) 68	Osteoporosis: 17.2 (15.4–19.2) Osteopenia: 17.2 (16.0–18.5) Normal: 16.7 (15.3–18.3) Geometric mean (95% CI)	BMD: Trochanter, intertrochanter, FN, Ward's triangle, TH [DXA, Hologic QDR-1000]	OR (95% CI) for mean BMD in relation to quartile categories of folate status + *P* for trend Category median (nmol/L): Q1: 8.0 Q2: 12.4 Q3: 20.3 Q4: 38.9	Q1: 1.1 Q2: 1.1 Q3: 1.5 Q4: 1.0 *P* for trend	(0.5–2.3) (0.0.5–2.9) (0.7–3.4) (reference) = 0.83^k^

Naharci et al. 2012 [38]	Cross-sectional Turkey *Moderate risk *	264 (100%) 77.0 ± 6.0	low (<7.0, group I): 0.0% borderline (7.0–10.9, group II): 9.2% normal (>10.9, group III): 90.8%	BMD: FN, TH, trochanter, intertrochanter [DXA, hologic QDR-4500]	Independent sample *t*-test for differences in FN BMD between group II and III of serum folate	No significant differences in BMD (all sites) between group II and III of folate status	

Ouzzif et al. 2012 [39]	Cross-sectional Morocco *Moderate risk *	188 (0%) 57.8 ± 8.5	15.6 ± 6.8	BMD: FN, LS, TH, trochanter [DXA, Lunar prodigy]	Multivariate regression, *β* for association folate-BMD *β* (SE) + *P* value	LS: 0.007 (0.002) *P* = 0.808^L^ TH: 0.006 (0.001) *P* = 0.834^L^	

Rumbak et al. 2012 [40]	Cross-sectional Croatia *Low risk *	131 (0%) 54.0 ± 4.9	22.4 ± 7.5	BMD: FN, LS, TH, radius [DXA, Lunar-prodigy]	Stepwise multivariate regression, *β* for association folate-BMD *β* + *P* value	Premenopausal: LS: 3.31 (4.73) *P* = 0.490^m, 2^ FN: 1.32 (4.90) *P* = 0.791^m, 2^ TH: 2.87 (4.35) *P* = 0.516^m, 2^ Postmenopausal: LS: −3.75 (3.47) *P* = 0.284^m, 2^ FN: −1.32 (3.15) *P* = 0.679^m, 2^ TH: 0.66 (3.89) *P* = 0.862^m, 2^	

*Serum/plasma folate concentrations were converted to nmol/L if applicable, using the following equation: 1 ng/ml = 2.266 nmol/L. Subsequent outcomes were also converted. Where possible, subgroups were combined. BMD sites—LS: Lumbar Spine, FN: Femoral Neck, TH: Total Hip ^#^data presented in article as *μ*mol/L, this is presumably a typing error and should be nmol/L.

^1^ *β* (SE) as calculated from data provided by author; ^2^ *β* (SE) as calculated from presented data.

^a^adjusted for age, BMI, smoking, coffee intake, physical activity, vit D use, educational level, estrogen use in women; ^b^adjusted for sex, age, height, weight, estrogen use in women; ^c^adjusted for age, gender, education, osteoporosis drug, serum creatinine, tHcy; ^d^Adjusted for duration of menopause, smoking, BMI, B_12_, tHcy; ^e^adjusted for age, BMI, logtHcy, logB_12_, creatinine clearance, smoking, alcohol intake;

^f^Adjusted for age, weight, weight change; ^g^Adjusted for smoking, BMI, creatinin, coffee intake, physical activity, use of estrogen therapy; ^h^adjusted for age, BMI, alkaline phosphatase; ^ i^adjusted for years since menopause, BMI, alkaline phosphatase, creatinine; ^j^adjusted for age, B_12_, tHcy, PTH, CTx, Ca, Cr; ^k^Adjusted for age, sex, ethnicity, BMI, smoking, physical activity, creatinin, alcohol, coffee, energy, calcium, vitamin D zinc intake; ^L^adjusted for age, BMI, tHcy, B_12_; ^m^adjusted for Age, BMI, smoking, alcohol, physical activity, tHcy, B_12_.

**Table 3:** Studies regarding the association between homocysteine and bone health.

Author Year	Study characteristics Duration of follow-up (when applicable) Country *Risk of bias *	Population characteristics: *N* (%men) Age (y) ± SD	Homocysteine status (*μ*mol/L) Mean ± SD	Outcome	Association type		Results	
Dhonukshe-Rutten et al. 2005 [3]	Cohort (3y) The Netherlands *High risk *	1253 (48%) 75.5 ± 6.6	geometric mean (10–90 percentile) ♀: 13.0 (8.6–19.7) ♂: 14.9 (10.2–22.8)	Fracture (verified by physician or radiograph)	*β* (SE) for association tHcy-fracture		♀: 0.07 (0.05)^a, 2^ ♂: 0.11 (0.05)^a, 2^	

Enneman et al. 2012 [30]	Cohort (7 y) The Netherlands *Moderate risk *	503 (0%) 68.5 (61.3–74.9) Median (range)	Median (range) 9.3 (3.5–29.7)	Fracture (verified by physician)	*β* (SE) for association tHcy-fracture		0.05 (0.02)^b, 2^	

Gerdhem et al. 2007 [29]	Cohort (7 y) Sweden *Low risk *	996 (0%) 75	Median (IQR) 14.1 (11.6–17.3)	Hip fracture (verified by radiograph)	*β* (SE) for association tHcy-hip fracture		0.07 (0.03)^c, 2^	

Gjesdal et al. 2007 [24]	Cohort (12.6 y) Norway *Low risk *	4761 (45%) 65–67 at baseline	♀: 11.6 ± 4.2 ♂: 13.1 ± 5.8	Hip fracture (verified by hospital discharge diagnoses)	*β* (SE) for association tHcy-hip fracture		♀: 0.05 (0.02)^d, 2^ ♂: 0.03 (0.03)^d, 2^	

Leboff et al. 2009 [28]	Nested case-control USA *Moderate risk *	800 (0%) 70.8 ± 6.2	11.2 ± 4.1	Hip fracture (verified by radiograph)	*β* (SE) for association tHcy-Hip fracture		0.07 (0.03)^e, 2^	

					HR (95% CI) for hip fracture risk by quartiles of tHcy. Mean tHcy per quartile:	
McLean et al. 2004 [4]	Cohort (♀ 15 y; ♂12.3 y) USA *Moderate risk *	1999 (41%) 70.0 ± 7.0	♀: 12.1 ± 5.3 ♂: 13.4 ± 9.1	Hip fracture (verified by review medical records)	♀: Q1: 7.6 ± 1.0 Q2: 9.9 ± 0.7 Q3: 12.2 ± 0.7 Q4: 18.6 ± 6.4	♂: 8.5 ± 0.9 11.0 ± 0.6 13.4 ± 0.9 20.8 ± 15.7	♀: 1: 1.00 (reference) 2: 0.78 (0.45–1.33) 3: 1.07 (0.64–1.78) 4: 1.92 (1.18–3.10)	♂: 1.00 (reference) 1.57 (0.54–5.14) 2.07 (0.70–6.09) 3.84 (1.38–10.70)
					HR (95% CI) for each increase of 1 SD in log-transformed tHcy concentration		♀/♂ Test for trend: *P* < 0.01 ♂ HR per SD 1.59 (1.31–1.94)^f^ ♀ HR per SD 1.26 (1.08–1.47)^f^	

McLean et al. 2008 [25]	Cohort (16 y) USA *Low risk *	979 (41%) 75.3 ± 4.9	73.7% normal (≤14 *μ*mol/l) 26.3% high (>14)	Hip fracture (verified by review medical records)	HR (95% CI) for high plasma tHcy (≥14 *μ*mol/L) versus normal tHcy		Normal 1.00 High 1.69	(reference) (1.12–2.55)^g^

Van Meurs et al. 2004 [5]	Cohort (4.7 y) The Netherlands *High risk *	2406 (47%) 73.9 ± 7.8	14.3 ± 5.8	Fracture (verified by physician)	RR (95% CI) for fracture for each increment of 1 SD in the natural log-transformed tHcy value.		1.4 (1.2–1.6)^h^	

Périer et al. 2007 [27]	Cohort (10 y) France *Moderate risk *	671 (0%) 61.6 ± 8.4	10.6 ± 3.5	Fracture (verified by radiograph or surgical report)	*β* (SE) for association tHcy-fracture		0.02 (0.02)^i, 3^	

Ravaglia et al. 2005 [26]	Cohort (4 y) Italy *Moderate risk *	702 (47%) 73.0 ± 6.0	Geometric mean (95% CI) 12.7 (11.3–15.1)	Fracture (verified by review medical records)	*β* (SE) for association tHcy-fracture		0.09 (0.05)^j, 2^	

Zhu et al. 2009 [31]	Cohort (5 y) Australia *Moderate risk *	1213 (0%) 75.2 ± 2.7	12.1 ± 4.6	Fracture (verified by radiograph)	*β* (SE) for association tHcy-fracture		−0.002 (0.006)^k, 2^	

Baines et al. 2007 [41]	Cross-sectional Great Britain *High risk *	328 (0%) 67.5 (40–85) mean (range)	12.3 ± 5.4	BMD: os calcis/heel bone [PIXI, GELunar]	Stepwise multivariate linear regression *β* (SE) + *P* value for association log tHcy-BMD		−1.548 (0.607) *P* = 0.011^L^	

Bozkurt et al. 2009 [32]	Cross-sectional Turkey *High risk *	178 (0%) 53.5 ± 8.0	10.4 ± 3.0^#^	BMD: FN/LS [DXA]	Logistic regression for FN, LS and FN + LS combined. *β* (SE) + *P* value for association hcy level under the median value-BMD		LS: −0.8 (0.5) *P* = 0.140 FN: −0.5 (0.6) *P* = 0.408 LS + FN: −1.3 (0.6) *P* = 0.032^m^	

Bucciarelli et al. 2010 [33]	Cross-sectional Italy *Moderate risk *	446 (0%) 65.1 ± 9.4	(geometric mean ± SD) 10.6 ± 1.3	BMD: FN, LS, TH [DXA, Prodigy, GE, Lunar]	Multivariate linear regression *β* for association log tHcy-total femur BMD. *β* (SE) *P* value		−0.050 (0.025) *P* = 0.048^n, 2^	

Cagnacci et al. 2008 [34]	Cohort Italy *Moderate risk *	117 (0%) 54.4 ± 0.5	(Mean ± SE) 10.7 ± 0.5	BMD: LS [DXA: Lunar DPX]	Regression analysis for Hcy-BMD change *β* (SE) + *P* value		−0.825 (1.09) *P* = 0.449^o^	

Cagnacci et al. 2003 [8]	Cross-sectional Italy *Moderate risk *	161 (0%) 53.3 ± 1.0	10.5 ± 0.9	BMD: LS [DXA: Lunar DPX]	Regression analysis, *β* for association Hcy-BMD		*β* = −0.002^p, 1^	

Gerdhem et al. 2007 [29]	Cohort (cross sect data) Sweden *Low risk *	996 (0%) 75	Median (IQR) 14.1 (11.6–17.3)	BMD: FN, LS, TH [DXA: Lunar DPX-L]	*t*-test for difference in BMD (*P* value) between highest quartile of hcy versus all others		FN: Q4 versus LS: Q4 versus TH: Q4 versus	Q1–3: *P* = 0.032 Q1–3: *P* = 0.821 Q1–3: *P* = 0.001

Gjesdal et al. 2006 [10]	Cross-sectional Norway *Moderate risk *	5329 (43%) middle aged: 47–50 Older: 71–75	♀: 10.2 ± 4.5 ♂: 11.8 ± 3.9	BMD: TH [DXA, Lunar EXPERT-XL]	Multivariate regression, *β* for association tHcy-BMD (*P* value) for middle aged and elderly women. (Data men not shown) OR (95% CI) for low BMD per category tHcy status + *P* for trend:		Mid. aged women: *β* = 0.004 (*P* < 0.001) elderly women: *β* = 0.003 (*P* < 0.001)^q^	
					1: <9.0 *μ*mol/L 2: 9.0–11.9 *μ*mol/L 3: 12.0–14.9 *μ*mol/L 4: ≥15 *μ*mol/L		♀: 1: 1.00 (reference) 2: 1.14 (0.90–1.44) 3: 1.30 (0.95–1.79) 4: 2.19 (1.48–3.25) *P* for trend <0.001	♂: 1.00 (reference) 1.01 (0.74–1.37) 1.12 (0.79–1.60) 1.02 (0.66–1.56) *P* for trend = 0.72^q^

Golbahar et al. 2004 [9]	Cross-sectional Iran *Moderate risk *	271 (0%) 60.8 ± 6.8	geometric mean (95% CI) 13.7 (7–14)	BMD: FN, LS [DXA, Lunar DPX-L]	*β* for association tHcy-BMD *β* (SE)		FN: −0.012 (0.023)^2^ LS: −0.010 (0.024)^2^	

Haliloglu et al. 2010 [36]	Cross-sectional Turkey *Moderate risk *	120 (0%) 54.4 ± 1.1	Osteoporotic: 15.0 ± 4.6 Osteopenic: 14.2 ± 3.7 Normal: 11.2 ± 2.6	BMD: LS [DXA, Lunar DPX-L]	ANOVA for difference in tHcy status per BMD group		tHcy was sign. higher in the osteoporotic group versus normal group (*P* < 0.05)	

Krivosikova et al. 2010 [37]	Cross-sectional Slovakia *High risk *	272 (0%) 41.3 ± 19.8	(*μ*mol/L) 14.6 ± 5.5	BMD: FN, LS, trochanter, TH [DXA, Lunar DPX-L]	Stepwise multivariate linear regression, *β* for association tHcy-BMD. *β* (SE) *P* value		FN: −0.093 (0.06) *P* = 0.100^r, 2^ LS: 0.003 (0.07) *P* = 0.965^r, 2^ TH: −0.134 (0.06) *P* = 0.033^r, 2^	

Morris et al. 2005 [7]	Cross-sectional USA *Low risk *	1550 (47%) 68	Osteoporosis: 11.5 (10.3–12.7) Osteopenia: 10.2 (9.5–10.8) Normal: 10.0 (9.6–10.5) Geometric mean (95% CI)	BMD: Trochanter, intertrochanter, FN, Ward's triangle, TH [DXA, Hologic QDR-1000]	OR (95% CI) for mean BMD in relation to quartile categories of tHcy status + *P* for trend Category median (*μ*mol/L): Q1: 6.9 Q2: 8.9 Q3: 10.8 Q4: 14.8		Q1: 1.0 (reference) Q2: 0.9 (0.4–1.9) Q3: 2.0 (0.7–5.1) Q4: 2.0 (0.8–4.9) *P* for trend = 0.09 ^s^Dose response analysis: subjects with tHcy level >20 *μ*mol/L had sign lower BMD than subj with tHcy level <10 *μ*mol/L	
							

Ouzzif et al. 2012 [39]	Cross-sectional Morocco *Moderate risk *	188 (0%) 57.8 ± 8.5	12.4 ± 4.1	BMD: FN, LS, TH, trochanter [DXA, Lunar prodigy]	Multivariate regression, *β* for association tHcy-BMD *β* (SE) + *P* value		LS: −0.089 (0.003)* P* = 0.200^t^ TH: −0.155 (0.002)* P* = 0.021^t^	

Périer et al. 2007 [27]	Cohort (cross-sect data) France *Moderate risk *	671 (0%) 61.6 ± 8.4	10.6 ± 3.5	BMD: FN, LS,TH [DXA, Hologic QDR-2000]	*β* for association tHcy-BMD *β* (SE)		LS: −0.000065 (0.004) FN: −0.006 (0.004) TH: −0.006 (0.004)^2^	

Rumbak et al. 2012 [40]	Cross-sectional Croatia *Low risk *	131 (0%) 54.0 ± 4.9	9.9 ± 2.0	BMD: FN, LS, TH, radius [DXA, Lunar-prodigy]	Stepwise multivariate regression, *β* for association tHcy-BMD. *β* (SE) for premenopausal and postmenopausal women		Premenopausal women^u, 2^: LS: 0.20 (0.14) *P* = 0.176 FN: 0.17 (0.15) *P* = 0.253 TH: 0.20 (0.14) *P* = 0.170 Postmenopausal women^u, 2^: LS: 0.12 (0.15) *P* = 0.439 FN: 0.20 (0.15) *P* = 0.181 TH: 0.12 (0.14) *P* = 0.391	

Zhu et al. 2009 [31]	Cohort (5 y) Australia *Moderate risk *	1213 (0%) 75.2 ± 2.7	12.1 ± 4.6	BMD: TH [DXA, Hologic Acclaim 4500A]	Change in hip BMD from 1 to 5 years per tertile of tHcy (*μ*mol/L) ANOVA		Tertile 1 and 3 differ significantly (*P* < 0.05)	

BMD sites—LS: Lumbar Spine, FN: Femoral Neck ^#^data presented in article as nmol/L, this is presumably a typing error and should be *μ*mol/L.

^1^data as provided by author on our request, ^2^ *β* (SE) as calculated from presented data, ^3^ *β* (SE) as calculated from data provided by author on our request.

^a^adjusted for age, BMI, smoking status, recurrent falling, serum creatinine; ^b^adjusted for age and BMI; ^c^adjusted for serum creatinine (natural log), B_12_ level, folic acid level, BMI, smoking, walking speed, BMD, LnPTH; ^d^adjusted for age, BMI, smoking, coffee intake, physical activity, vit D use, educational level, estrogen use in women; ^e^case-control matched for age and ethnicity. Adjusted for BMI, parental history of hip fracture, treated diabetes, alcohol use, smoking, history of stroke, total calcium intake; ^f^adjusted for sex, age, height, weight, smoking status, caffeine intake, alcohol intake, education level, estrogen use in women; ^g^adjusted for sex, age, height, weight, estrogen use in women; ^h^adjusted for age, sex, BMI, changes in BMI before entry in the study, smoking, fall history, serum creatinine; ^i^adjusted for age, prevalent fractures, BMD, calcium intake, physical activity, vitamin D level, creatinine, albumin, estradiol; ^j^adjusted for age, gender, education, serum creatinine, osteoporosis drugs; ^k^adjusted for age, weight, hip BMD, prevalent fracture, calcium treatment; ^L^adjusted for weight, cysteine, smoking and height;^ m^Adjusted for duration of menopause, smoking, BMI, folic acid levels, homocysteine levels;^ n^adjusted for age, BMI, logFolate, logB_12_, creatinine clearance; ^o^Adjusted for age, weight, weight change; ^ p^Adjusted for BMI, smoking, age;^ q^Adjusted for smoking, BMI, creatinine, coffee intake, physical activity, use of estrogen therapy; ^r^adjusted for age, B_12_, folate, PTH, CTx, Ca, Cr; ^s^adjusted for age, sex, ethnicity, BMI, smoking, physical activity, creatinin, alcohol, coffee, energy, calcium, vitamin D zinc intake; ^t^adjusted for age, BMI, folate, B_12_; ^u^adjusted for age, BMI, smoking, alcohol intake, physical activity, duration of menopause, HRT, levels of hcy, vitB_12_ and folate.
