# Supplementary material for: Vitamin B12, Folate, Homocysteine, and Bone Health in Adults and Elderly People: A Systematic Review with Meta-Analyses
Source: J Nutr Metab. 2013 Feb 20;2013:486186. doi: 10.1155/2013/486186 (PMC3590816; doi:10.1155/2013/486186)
Supplement: Supplementary file 1 — The electronic databases MEDLINE, EMBASE, and Cochrane Library Central were searched, using search terms in “MeSH” terms and “title” and “abstract” on study designs in humans, vitamin B12, folate, homocysteine, and intake or status. The fullMedline search strategy is available in Appendix 1. [file 486186.f1.docx]

|  | **Search term** |
| --- | --- |
| 1 | Randomized controlled trial.pt. |
| 2 | Controlled clinical trial.pt. |
| 3 | Randomized.ab. |
| 4 | Placebo.ab. |
| 5 | Randomly.ab. |
| 6 | Clinical trials as topic.sh. |
| 7 | Trial.ab. |
| 8 | Randomised.ab. |
| 9 | 6 or 3 or 7 or 8 or 2 or 1 or 4 or 5 |
| 10 | (animals not (human and animals)).sh. |
| 11 | 9 not 10 |
| 12 | (cohort* or “case control$” or cross-sectional$ or “cross sectional” or case-control$ or prospective or “systematic$ review$”).mp. [mp=title, original title, abstract, name of substance word, subject heading word] |
| 13 | Exp meta-analysis/ or exp multicenter study/ or follow-up studies/ or prospective studies/ or intervention studies/ or epidemiologic studies/ or case-control studies/ or exp cohort studies/ or longitudinal studies/ or cross-sectional studies/ |
| 14 | 12 or 13 |
| 15 | 14 not 10 |
| 16 | 15 or 11 |
| 17 | ((“vitamin b12” or vitamin-b12 or “vitamin b 12” or “vitamin-b 12” or cobalamin$ or cyanocobalamin$ or hydroxocobalamin$ or methylcobalamin* or adenosylcobalamin*) adj5 (intake* or diet* or supplement$ or deplet$ or status or serum or plasma or “methylmalonic acid” or MMA or methylmalonate or “propanedioic acid” or “methylpropanedioic acid” or “malonic acid” or Holotranscobalamin$ or holo-transcobalamin$ or holoTC or holo-tc or concentration$ or expos$ or fortif$)).ti,ab. |
| 18 | Nutritional support/ or Dietary supplements/ or nutritional requirements/ |
| 19 | Exp Nutritional Status/ or exp Deficiency Diseases/ or supplementation/ or diet supplementation/ or dietary intake/ or exp diet therapy/ or Diet/ or Food, Fortified/ or nutrition assessment/ or Nutritive Value/ |
| 20 | (intake$ or diet$ or supplement$ or deplet$ or status or serum or plasma or “methylmalonic acid” or MMA or methylmalonate or “propanedioic acid” or “methylpropanedioic acid” or “malonic acid” or Holotranscobalamin$ or holo-transcobalamin$ or holoTC or holo-TC or concentration$ or expos$ or fortif$).ti,ab. |
| 21 | Methylmalonic acid/ |
| 22 | 18 or 19 or 20 or 21 |
| 23 | Hydroxocobalamin/ |
| 24 | Vitamin b 12/ |
| 25 | 23 or 24 |
| 26 | 25 and 22 |
| 27 | Vitamin b 12 deficiency/ |
| 28 | 17 or 26 or 27 |
| 29 | 28 and 16 |

**Appendix 1: Search strategies in Medline**

Vitamin B12:

|  | **Search term** |
| --- | --- |
| 1 | Randomized controlled trial.pt. |
| 2 | Controlled clinical trial.pt. |
| 3 | Randomized.ab. |
| 4 | Placebo.ab. |
| 5 | Randomly.ab. |
| 6 | Clinical trials as topic.sh. |
| 7 | Trial.ab. |
| 8 | Randomised.ab. |
| 9 | 6 or 3 or 7 or 8 or 2 or 1 or 4 or 5 |
| 10 | (animals not (human and animals)).sh. |
| 11 | 9 not 10 |
| 12 | (cohort* or “case control$” or cross-sectional$ or “cross sectional” or case-control$ or prospective or “systematic$ review$”).mp. |
| 13 | Exp meta-analysis/ or exp multicenter study/ or follow-up studies/ or prospective studies/ or intervention studies/ or epidemiologic studies/ or case-control studies/ or exp cohort studies/ or longitudinal studies/ or cross-sectional studies/ |
| 14 | 12 or 13 |
| 15 | 14 not 10 |
| 16 | 15 or 11 |
| 17 | (homocysteine or s-adenosylhomocysteine or Hcy or tHcy or homo-cysteine or homo-cys).ti,ab. |
| 18 | Homocysteine/ |
| 19 | 17 or 18 |
| 20 | 19 and 16 |
| 21 | Bone$ or skelet$ or BMD or BMC or BMDD or oste$ or ossi$ or osse$ or fracture$.mp. |
| 22 | 20 and 21 |

Homocysteine

Folate RCTs

|  | **Search term** |
| --- | --- |
| 1 | infant nutrition/ or artificial milk/ or breast milk/ or bottle feeding/ or breast feeding/ or lactation/ |
| 2 | random*.ti,ab. |
| 3 | factorial*.ti,ab. |
| 4 | (crossover* or cross over* or cross-over*).ti,ab. |
| 5 | placebo*.ti,ab. |
| 6 | (doubl* adj blind*).ti,ab. |
| 7 | (singl* adj blind*).ti,ab. |
| 8 | Crossover Procedure/ |
| 9 | Double Blind Procedure/ |
| 10 | Randomized Controlled Trial/ |
| 11 | Single Blind Procedure/ |
| 12 | animal/ or nonhuman/ or animal experiment/ |
| 13 | human/ |
| 14 | (folate or "folic acid" or (vitamin adj5 B9)).ti,ab. |
| 15 | methylenetetrahydrofolic acid/ or 5 methyltetrahydrofolic acid/ or tetrahydrofolic acid/ or tetrahydrofolic acid derivative/ |
| 16 | (intake* or diet* or supplement* or deplet* or status or concentration* or expos* or fortif* or plasma or serum or "red blood cell*" or erythrocyte or RBC or RCF).ti,ab. |
| 17 | ((folate or "folic acid" or (vitamin adj5 B9)) adj3 (intake* or diet* or supplement* or deplet* or status or concentration* or expos* or fortif* or plasma or serum or "red blood cell*" or erythrocyte or RBC or RCF)).ti,ab. |
| 18 | folic acid blood level/ |
| 19 | Folic Acid Deficiency/ |
| 20 | supplementation/ or diet supplementation/ or dietary intake/ or exp diet restriction/ or exp food intake/ |
| 21 | exp nutritional status/ or nutritional deficiency/ or vitamin blood level/ |
| 22 | *Homocysteine/ |
| 23 | (homocysteine or hcy).ti,ab. |
| 24 | 6 or 11 or 3 or 7 or 9 or 2 or 8 or 4 or 10 or 5 |
| 25 | 13 and 12 |
| 26 | 12 not 25 |
| 27 | 24 not 26 |
| 28 | Folic Acid/ |
| 29 | 28 or 14 |
| 30 | 22 or 21 or 23 or 16 or 20 |
| 31 | 1 or 30 |
| 32 | 30 and 29 |
| 33 | 32 or 17 or 15 |
| 34 | 27 and 33 |
| 35 | 31 and 29 |
| 36 | 35 or 17 or 15 |
| 37 | 27 and 36 |

Folate – Cross-sectional study design only

|  | **Search term** |
| --- | --- |
| 1 | infant nutrition/ or artificial milk/ or breast milk/ or bottle feeding/ or breast feeding/ or lactation/ |
| 2 | (folate or "folic acid" or (vitamin adj5 B9)).ti,ab. |
| 3 | methylenetetrahydrofoli  acid/ or 5 methyltetrahydrofolic acid/ or tetrahydrofolic acid/ or tetrahydrofolic acid derivative/ |
| 4 | (intake* or diet* or supplement* or deplet* or status or concentration* or expos* or fortif* or plasma or serum or "red blood cell*" or erythrocyte or RBC or RCF).ti,ab. |
| 5 | ((folate or "folic acid" or (vitamin adj5 B9)) adj3 (intake* or diet* or supplement* or deplet* or status or concentration* or expos* or fortif* or plasma or serum or "red blood cell*" or erythrocyte or RBC or RCF)).ti,ab. |
| 6 | folic acid blood level/ |
| 7 | Folic Acid Deficiency/ |
| 8 | supplementation/ or diet supplementation/ or dietary intake/ or exp diet restriction/ or exp food intake/ |
| 9 | exp nutritional status/ or nutritional deficiency/ or vitamin blood level/ |
| 10 | *Homocysteine/ |
| 11 | (homocysteine or hcy).ti,ab. |
| 12 | Folic Acid/ |
| 13 | 12 or 2 |
| 14 | 10 or 9 or 11 or 4 or 8 |
| 15 | 1 or 14 |
| 16 | 15 and 13 |
| 17 | 16 or 5 or 3 |
| 18 | ("cross sectional*" or cross-sectional*).mp. [mp=title, abstract, subject headings, heading word, drug trade name, original title, device manufacturer, drug manufacturer name] |
| 19 | cross-sectional studies/ |
| 20 | 18 or 19 |
| 21 | 20 and 17 |

Folate – Cohorts

|  | **Search term** |
| --- | --- |
| 1 | infant nutrition/ or artificial milk/ or breast milk/ or bottle feeding/ or breast feeding/ or lactation/ |
| 2 | (folate or "folic acid" or (vitamin adj5 B9)).ti,ab. |
| 3 | methylenetetrahydrofolic acid/ or 5 methyltetrahydrofolic acid/ or tetrahydrofolic acid/ or tetrahydrofolic acid derivative/ |
| 4 | (intake* or diet* or supplement* or deplet* or status or concentration* or expos* or fortif* or plasma or serum or "red blood cell*" or erythrocyte or RBC or RCF).ti,ab. |
| 5 | ((folate or "folic acid" or (vitamin adj5 B9)) adj3 (intake* or diet* or supplement* or deplet* or status or concentration* or expos* or fortif* or plasma or serum or "red blood cell*" or erythrocyte or RBC or RCF)).ti,ab. |
| 6 | folic acid blood level/ |
| 7 | Folic Acid Deficiency/ |
| 8 | supplementation/ or diet supplementation/ or dietary intake/ or exp diet restriction/ or exp food intake/ |
| 9 | exp nutritional status/ or nutritional deficiency/ or vitamin blood level/ |
| 10 | *Homocysteine/ |
| 11 | (homocysteine or hcy).ti,ab. |
| 12 | Folic Acid/ |
| 13 | 12 or 2 |
| 14 | 10 or 9 or 11 or 4 or 8 |
| 15 | 1 or 14 |
| 16 | 15 and 13 |
| 17 | 16 or 5 or 3 |
| 18 | cohort*.mp. [mp=title, abstract, subject headings, heading word, drug trade name, original title, device manufacturer, drug manufacturer name] |
| 19 | cohort studies/ |
| 20 | 18 or 19 |
| 21 | 20 and 17 |
